# A New Defence for Murder

**Published:** 1869-03-15

**Authors:** 


### EDITORIAL.

### *A New Defense for Murder.*

There are perhaps few of our readers who have not been by turns amused, disgusted and wearied at the frequency with which, of late years, the plea of insanity has been urged as an excuse for crime. Nothing appears so suggestive of the existence of insanity previously unsuspected as the perpetration of some outrage upon society, and nothing so refreshing to the memories of witnesses upon the subject of hereditary taints. It behooves the Thugs and cut-throats to overhaul their genealogical registers and to trace, if possible, their relationship to some crack-brained ancestor, some lunatic aunt, or delirious uncle, whose possible bequest of his malady may prove an inestimable treasure: more precious than fine gold, or even five-twenties; for it may save his (or her) neck from a halter. It becomes a question for the consideration of parents, whether a legacy of delirium tremens or epilepsy be not quite as valuable an inheritance for their children as a life insurance policy. It certainly has affected many times what the latter never has—insured life. But our cousins, the other doctors (of the law) appear to be widening their field of philanthropy. Some individual in haste to get rich, commits a forgery, and he is straightway found guilty —not of crime—but of moral insanity. A woman shoots her lover (or some other woman's) and she is found guilty of menstrual irregularity. Again, a man shoots his employer, and threatens to shoot others, and he is found guilty of epilepsy. We do not discover, however, that any means have been taken to prevent a repetition of these diverting eccentricities of genius, their perpetrators decided by verdicts of juries to be legally irresponsible agents, being still permitted to go at large. And as the class exempted from legal responsibility threatens to become so large that it cannot by any possibility be expelled from society, would it not be well for the Legislature to provide a sane asylum for the reception and protection of all those who desire neither to kill nor yet to be killed. The abolition of the death penalty is "a consummation most devoutedly to be wished," and should be effected just as soon as the assassins set the example.

* * * ILL., March 2, 1869.

EDITOR MEDICAL JOURNAL:

There are "physicians" in Chicago advertising for partners with "a small capital." These "physicians" hold out dazzling inducements to young graduates, alleging that their business brings them several thousands of dollars each year; that the new partner will be received on equal shares, by paying $1,000 or $1,500 down. That they are regulars, and are called in consultation with members of Rush College Faculty frequently, etc. Their books are open to inspection, and show a stunning array of cash receipts. Please allow me to say they (the "physicians") are *rascals*, and that any young physicians who pay their money to such men will be swindled. The graduates from Rush are, many of them, seeking openings, and will be drawn to Chicago by these tempting advertisements, and taken in. Most, if not all, of these graduates take your JOURNAL. Dr. * * told me I better call your attention to this matter, and ask you to state briefly in your next issue that these advertising men are *swinders*, and then none of our boys will be defrauded by such pests. Yours, truly

It is scarcely necessary for THE JOURNAL to say more than has been well said by our correspondent. We have written scores of private letters to parties inquiring of us, denouncing these scamps as they deserve. It is a professional *confidence game*, and richly entitles its practitioners to the penitentiary.

